# Platelet activation and prothrombotic properties in a mouse model of peritoneal sepsis

**DOI:** 10.1038/s41598-018-31910-8

**Published:** 2018-09-10

**Authors:** Fanny Vardon Bounes, Vincent Mémier, Marina Marcaud, Aemilia Jacquemin, Hind Hamzeh-Cognasse, Cédric Garcia, Jennifer Series, Pierre Sié, Vincent Minville, Marie-Pierre Gratacap, Bernard Payrastre

**Affiliations:** 1 INSERM, U1048 et Université Toulouse III, Institut des Maladies Métaboliques et Cardiovasculaires (I2MC), Toulouse, 31400 France; 2 Anesthesiology and Critical Care Unit, Centre hospitalier universitaire de Toulouse, Toulouse, 31400 France; 3 Haematology laboratory, Centre hospitalier universitaire de Toulouse, Toulouse, 31400 France; 4 EA3064-GIMAP, Université de Lyon, Saint-Etienne, 42023 France

## Abstract

Sepsis is associated with thrombocytopenia and microvascular thrombosis. Studies have described platelets implication in this pathology but their kinetics of activation and behavior remain poorly known. We show in a mouse model of peritonitis, the appearance of platelet-rich thrombi in organ microvessels and organ damage. Complementary methods are necessary to characterize platelet activation during sepsis as circulating soluble markers and platelet-monocyte aggregates revealed early platelet activation, while surface activation markers were detected at later stage. A microfluidic based *ex-vivo* thrombosis assay demonstrated that platelets from septic mice have a prothrombotic behavior at shear rate encountered in microvessels. Interestingly, we found that even though phosphoinositide-3-kinase β−deficient platelet mice formed less thrombi in liver microcirculation, peritoneal sepsis activates a platelet alternative pathway to compensate the otherwise mandatory role of this lipid-kinase to form stable thrombi at high shear rate. Platelets are rapidly activated during sepsis. Thrombocytopenia can be attributed in part to platelet-rich thrombi formation in capillaries and platelet-leukocytes interactions. Platelets from septic mice have a prothrombotic phenotype at a shear rate encountered in arterioles. Further studies are necessary to unravel molecular mechanisms leading to this prothrombotic state of platelets in order to guide the development of future treatments of polymicrobial sepsis.

## Introduction

Sepsis is a major cause of mortality and critical illness in the world^1,2^ and is considered as a major public health concern whose incidence is increasing^3^. Severe sepsis is defined as life-threatening organ dysfunction caused by a dysregulated host response to infection^4^.

It is now well documented that the role of platelets includes an immune response function during the host response to infections^5,6^. Platelets are thought to play a major role in sepsis with thrombocytopenia being recognized as an independent risk factor for mortality of patients admitted to the intensive care units with severe sepsis or septic shock^7^. Platelets are anucleated circulating cells playing an essential role in hemostasis and thrombosis. They are highly reactive to extracellular stimuli through activation of a variety of specific membrane receptors for soluble agonists or adhesive proteins allowing platelet adhesion, activation, secretion and aggregation to form a plug, which, together with activation of the coagulation system, safeguards vessel integrity and prevent hemorrhage^8^. Under conditions of high shear, the initial recruitment of platelets to an injured vessel wall is mediated by the reversible interaction with von Willebrand factor (VWF) that binds to collagen fibers of the subendothelial matrix and the platelet receptor glycoprotein GPIb. This interaction allows platelets to establish contacts with collagen leading to their activation and the release of secondary mediators such as adenosine diphosphate (ADP) and thromboxane A_2_ (TxA_2_). These mediators, together with thrombin generated by the coagulation cascade, further activate platelets leading to their aggregation and the stabilization of the platelet-rich thrombus^9^. Sepsis is a multistep disease in which platelets are implicated through several mechanisms including recruitment of immune cells. This recruitment contributes to a hyper-inflammatory state^10^ with subsequent development of microvascular occlusive syndromes and thus worsening multiple organ failure^11,12^. The procoagulant state in sepsis and formation of neutrophils extracellular DNA traps (NETs) and microthrombi as a defense strategy increase the risk of vaso-occlusive complications^13^. It is important to note that at certain stages of sepsis platelets have a protective role through tempering macrophage-dependent inflammation^14^ and limiting clinical severity through the podoplanin-CLEC2 axis^15^.

Several reports suggest that platelets may be a relevant therapeutic target in sepsis. *In vitro* studies have described bacterial-induced platelet activation processes and identified different mechanisms of interactions between platelets and bacteria^16–19^. Evidence is accumulating that inhibition of platelet function can modulate inflammatory markers^20^. Drugs inhibiting platelet activation, such as acetylsalicylic acid (ASA) or P2Y12 inhibitors, may have a benefit in reducing thrombo-inflammation, arterial microthrombi and in turn multiple organ failure in critically ill septic patients. Furthermore, new targets for antithrombotic therapy have been proposed such as Class IA phosphoinositide 3-kinase (PI3K) β isoform that participates in the regulation of a range of functional platelet responses, including sustained activation of α_IIb_β_3_ integrin. It has been shown that *in vivo*, isoform-selective PI3Kβ inhibitors prevent occlusive thrombus formation but do not prolong the bleeding time^21,22^. Such inhibitor could be of interest in the treatment of septic patients. However, it is essential to get further insights on the time course of platelet activation during sepsis, on the consequences of sepsis on platelet prothrombotic properties at arterial shear rate and to evaluate the impact of platelet PI3Kβ inhibition. Only a few studies have investigated platelet activation during sepsis in humans^13,23–26^ or in experimental animal models^27,28^. Recently, a study of platelet activation dynamics during the progression of a *streptococcus pyogenes* infection has proposed that monitoring platelet activation may provide prognostic information in this type of sepsis^29^.

The aim of the present work was to characterize platelet activation during the development of a cecal ligation and puncture (CLP) model of polymicrobial peritoneal sepsis in mice. Furthermore, we studied platelet prothrombotic properties at high shear rate encountered in microvessels and evaluated the impact of platelet PI3Kβ inhibition.

## Results

### Characterization of sepsis and platelet parameters over time in the CLP mice model

**Figure 1:**
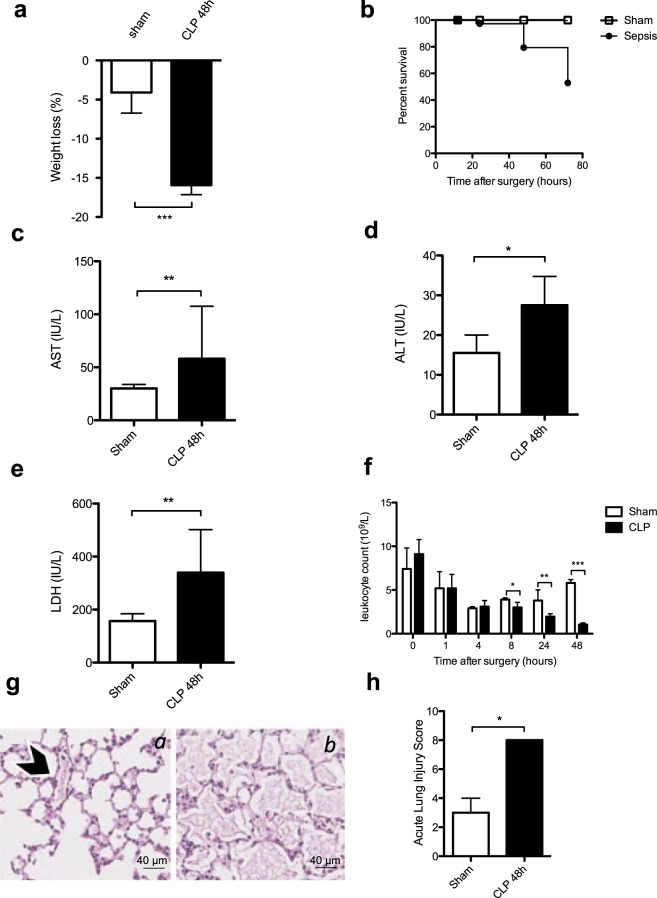
Characterization of sepsis after cecal ligation and puncture. (**a**) Weight loss was increased 48 hours post procedure in the CLP group of mice (black bar) compared to the sham group (white bar). Results are expressed as percentage of weight loss and are median [25–75^th^ percentiles] (n = 14, ***p < 0.001). (**b**) Survival was quantified at 72 hours post CLP. At 72 h, the overall mortality was 47% in CLP group. Results are expressed as percentage of survival (n = 36, *p* < 0.05). Biochemical analysis were performed with a PENTRA 400 ABXc analyzer for aspartate aminotransferase (AST) (**c**), alanine aminotransferase (ALT) (**d**) and lactate dehydrogenase (LDH) (**e**). Results are presented as median [25–75^th^ percentiles] (n = 6, *p < 0.05, **p < 0.01, ***p < 0.001). **(f)** Leukocyte count was measured 48 h post surgery and compared in sham versus CLP group. Results are expressed as median [25–75^th^ percentiles] (n = 6 to 30 *p < 0.05,**p > 0.001, ***p < 0.0001). **(g)** Representative images of lung sections stained with hematoxylin and eosin 48 h post surgery. The arrowhead shows a blood vessel section which integrity is conserved in a sham-operated mouse (**a**). In the CLP group of mice (*b*) important alveolar injuries are observed as quantified by the Acute Lung Injury (ALI) Score 48 h post CLP induction (**h**). Results are median ± IQR of 7 independent experiments (*p < 0.05) and representative images are shown (**g**).

To analyze sepsis development and progression in our mouse model of peritonitis a series of biological parameters were measured at different time points after CLP (Fig. 1). There was significant weight loss in the CLP group (sham −4.1 [−6.7–3.0] % versus CLP −15.9 [−17.2–−11.9] %) accompanied by an overall mortality of 47% three days after surgery (Fig. 1a,b). The plasma levels of IL-1β, TNFα and IL-6 were significantly increased in the CLP group after 6 hours (Suppl Fig. 1) indicating a pro-inflammatory stage rapidly generated following CLP. A marked elevation of liver transaminases AST and ALT was measured in the CLP group (sham AST 30.0 [25.8–33.8] IU/L versus CLP AST 58.0 [48.5–107.5] IU/L, n = 6, *p* = 0.005 and sham ALT 15.5 [10–20] IU/L versus CLP ALT 27.5 [20.3–34.8] IU/L, n = 6, *p* = 0.019) indicating liver cytolysis (Fig. 1c,d). Lactate dehydrogenase (LDH) was also significantly increased in the CLP group (Fig. 1e). There was no difference in serum creatinine level measured between the 2 groups (Suppl Fig. 1d). Analysis of these selected biochemical parameters indicated organ cytolysis 48 hours after CLP.

There was a significant leukocyte count decrease in the CLP group after 8 hours that persisted until 48 hours of sepsis (5.8 [4.7–6.2] 10^9^/L in sham group versus 1.05 [0.8–1.23] 10^9^/L in CLP group, (n = 30), *p* < 0.0001) (Fig. 1f). Lymphocyte and monocyte counts were particularly low 48 hours after the surgery while neutrophil count declined significantly 4 hours after CLP (Suppl Fig. 2a–c).

Histological assessment of lung sections revealed significant inflammatory infiltrates as demonstrated by interalveolar thickening, interstitial edema and a significantly elevated mean acute lung injury score 48 hours after CLP (Fig. 1g,h). Neutrophil infiltration in both lung and liver was increased in the CLP group (Suppl Fig. 3a).

**Figure 2:**
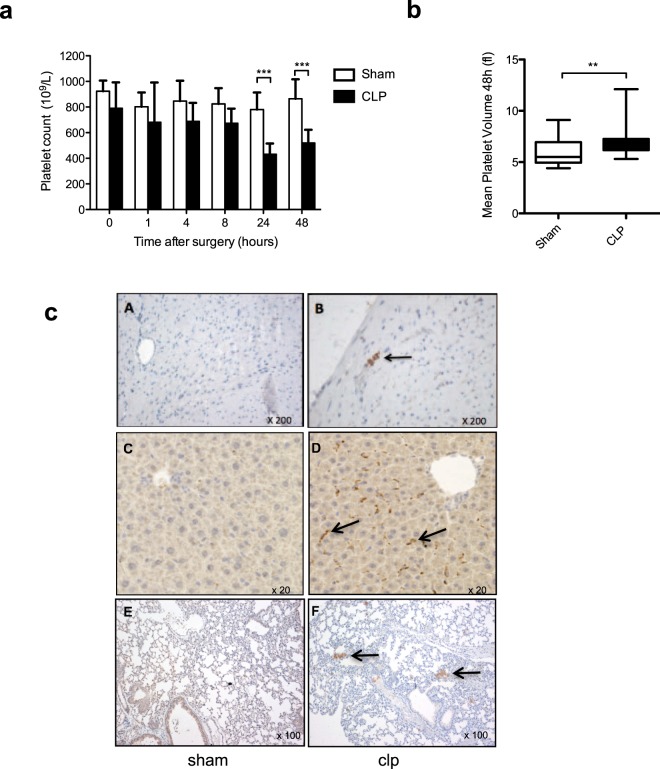
Sepsis promotes thrombocytopenia and thrombus formation in lung capillaries after cecal ligation and puncture. (**a**) Whole blood platelet count kinetics at 48 h post CLP surgery. Results are expressed as platelets x 10^9^/L and are median ± IQR of 30 independent experiments (**p < 0.01, ***p < 0.001). **(b)** Comparison of Mean Platelet Volume (MPV) 48 h post CLP surgery. Whisker boxes are constructed as follow: min, max, median, 25–75th percentiles (n = 30, **p < 0.01). (**c**) Representative histological sections of heart (A,B), liver (C,D) and lung (E,F) tissues 48 h post surgery. Sections from CLP (B,D,F) or sham-operated animals (A,C,E) as controls were stained with Masson’s trichrome and platelets were specifically labeled with an anti-αIIb antibody. Arrows highlight platelet-rich thrombi in microvessels. Images (x20, x100 and x200 magnification) shown are representative of 3 independent experiments.

As shown in Fig. 2A, we observed a progressive platelet count decrease in the CLP group that became significant after 24 hours (sham 780 [657–913] 10^9^/L versus CLP 431 [380–515] 10^9^/L, n = 15, *p* < 0.0001) and persisted two days post CLP (sham 864 [785–1016] 10^9^/L versus CLP 519 [393–622] 10^9^/L, n = 30, *p* < 0.0001). A significant increase in mean platelet volume was also observed at 48 hours suggesting occurrence of platelet renewal (sham 5.5 [5.0–7.0] fl versus CLP 6.9 [6.2–7.3] fl, n = 30, *p* = 0.001) (Fig. 2b). Interestingly, the circulating platelet count decrease in the CLP group correlated with the appearance of platelet-rich thrombi in tissue microvessels as shown by immunohistopathology (Fig. 2c). Thrombi could be observed in the heart (upper panels), in periportal capillaries (middle panels), and in pulmonary microvessels (lower panels) of the CLP group of mice while no thrombus formation could be seen in the sham group (Fig. 2c).

Of note, identification of bacteria from blood cultures obtained 48 hours after CLP indicated the presence of *enterococcus faecalis*, and of *citrobacter braakii* (Suppl Fig. 3b).

### Kinetics of platelet activation during sepsis

To investigate the level and kinetics of platelet activation during sepsis we analyzed platelet intrinsic markers including membrane exposure of CD62P and fluorescently labeled fibrinogen binding to activated GpIIbIIIa (α_IIb_β3), formation of heterotypic aggregates between platelets and leukocytes and soluble markers in plasma such as soluble CD40L (sCD40L) and eicosanoids.

**Figure 3:**
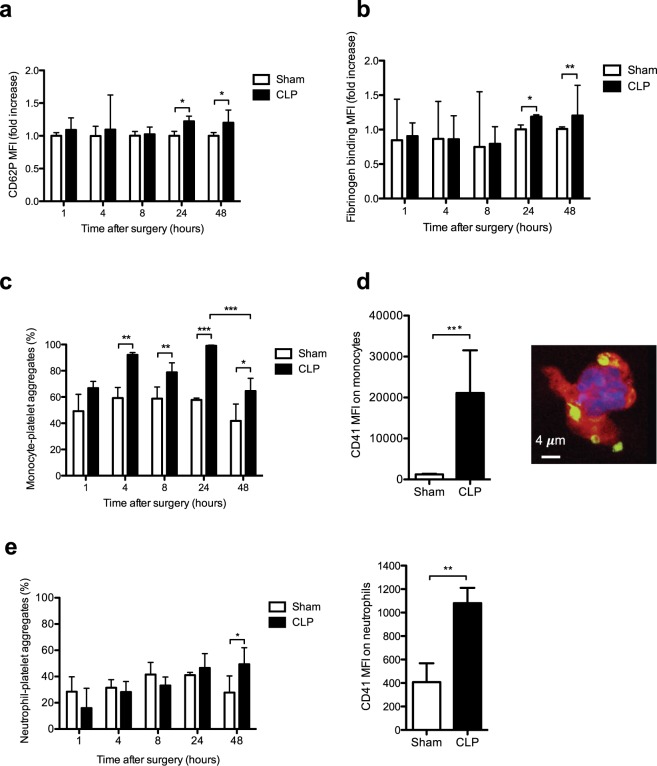
Expression of surface platelet activation markers and elevation of leukocyte-platelets interactions during sepsis. (**a**) Expression of the surface platelet activation marker CD62P analyzed by flow cytometry during sepsis. (**b**) Activation of α_IIb_β_3_ (GpIIbIIIa) integrin at the platelet surface assessed by oregon green fibrinogen binding and flow cytometry analysis. Results are expressed as median fluorescence intensity and are median fold increase ± IQR of 6 to 8 independent experiments (*p < 0.05, **p < 0.01). (**c**) Whole blood monocyte-platelet aggregates quantified at different times post surgery in sham and CLP-operated mice. Results are expressed as percentage of monocyte-platelet aggregates and are median ± IQR of 4 to 6 independent experiments (*p < 0.05, **p < 0.01, ***p < 0.001). (**d**) Density of platelets per monocytes. The MFI values of the platelet marker (CD41) on monocytes was measured 24 h after CLP by flow cytometry to evaluate the platelet density per monocyte (left panel). After sorting by flow cytometry the platelet-monocyte aggregates were spin down onto poly-lysine coated slides and observed by confocal microscopy (right panel). A representative confocal image is show to illustrate the interaction of platelets (CD41, green) and monocyte (CD115, red) 24 h post CLP. The monocyte nucleus was labeled with DAPI (blue). (**e**) Whole blood neutrophil-platelet aggregates quantified at different times after surgery in sham and CLP-operated mice. Results are expressed as percentage of neutrophil-platelet aggregates and are median ± IQR of 3 to 7 independent experiments (*p < 0.05) (left panel). The MFI values of the platelet marker (CD41) on neutrophils was measured 48 h after CLP to evaluate the platelet density per neutrophil (right panel).

P-selectin (CD62P) exposure at the platelet surface assessed by flow cytometry at intervals following CLP indicated a significant platelet activation 24 hours after CLP that persisted at 48 hours (Fig. 3a). Consistent with these results, fibrinogen fixation on activated GpIIbIIIa was significantly enhanced 24 hours after CLP and further increased at 48 hours (Fig. 3b). However, activated platelets may be rapidly removed from the circulation and/or conjugated to leukocytes^30^. As shown in Fig. 3c, as soon as 4 hours monocyte-platelet interactions significantly increased with a maximal increase 24 hours after surgery. In spite of an important decline of circulating monocytes (Suppl Fig. 2b), platelet-monocyte aggregates were still significantly elevated 48 hours after CPL. Figure 3d is a representative confocal image showing that several platelets interacted with monocytes 24 hours after CLP. Interestingly, at that time point, the density of platelets per monocyte, estimated by median fluorescence intensity of the platelet marker CD41^31^, was strongly increased following sepsis compared to Sham operated mice (Fig. 3d). Of note, the formation kinetics of neutrophil-platelet aggregates formation were different. Indeed, these heterotypic aggregates were observed later in the sepsis and were significantly increased 2 days after surgery with an increase in the density of platelets per neutrophil compared to Sham mice (Fig. 3e, right panel).

**Figure 4:**
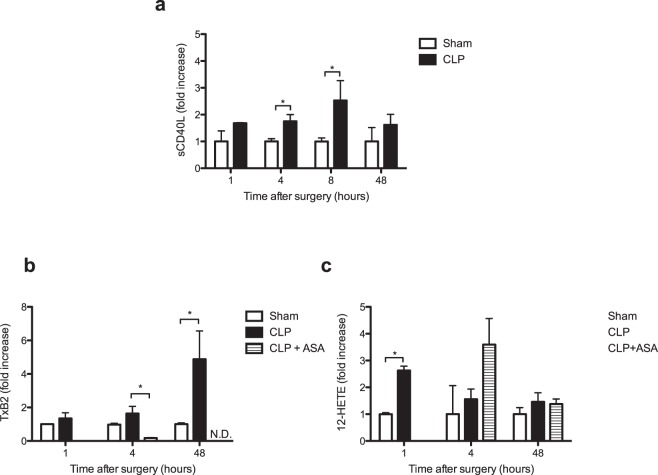
Early elevation of soluble markers of platelet activation during sepsis. (**a**) Levels of plasma soluble CD40L (sCD40L) and eicosanoids at different times in sham and CLP mice. Results are expressed as fold increase and are median (25–75^th^ percentile) of 4 to 7 independent experiments (*p < 0.05). (**b**) Kinetics of TxB2, the stable metabolite of TxA2, and (**c**) 12-HETE production in plasma of sham, CLP-operated mice and CLP-operated mice treated with aspirin. The quantification was performed by a lipidomics LC-MS/MS technique. Results are expressed as fold increase and are median (25–75^th^ percentile) of 3 to 6 independent experiments (*p < 0.05, **p < 0.01). N.D., not detectable.

Analysis of soluble markers of platelet activation, such as plasmatic sCD40L and eicosanoids, provides the opportunity to detect low grade circulating platelet activation or platelet activation even if activated platelets are no longer circulating (i.e. bound to the endothelium) or in complex with leukocytes^32^. CLP significantly increased plasma levels of sCD40L as soon as 4 hours (1.75 [1.48–1.99] fold increase *n* = 3, *p* = 0.04) and 8 hours (2.53 [1.66–3.27] fold increase, *n* = 4, *p* = 0.006) after surgery (Fig. 4a). This increase was no longer significant 2 days after CLP.

Eicosanoids are locally acting bioactive signalling lipids derived from arachidonic acid and related polyunsaturated fatty acids that regulate a diverse set of homeostatic and inflammatory processes^33^. After activation, platelets produce eicosanoids via cyclooxygenase and lipooxygenase pathways, particularly thromboxane A2 (TxA2) and 12-hydroeicosatetraeinoic acid (12-HETE). Thromboxane B2 (TxB2), the stable metabolite of TxA2, increased 4 hours after CLP to become significantly more abundant after 48 hours in the CLP group (Fig. 4b). As a control, inhibition of cyclooxygenase by aspirin treatment of mice fully inhibited TxB2 production following CLP. The lipoxygenase product 12-HETE was also rapidly produced, measured 1 hour after CLP its plasma concentration was significantly elevated compared to the sham group of mice (Fig. 4c). In this case, as expected, inhibition of cyclooxygenase by aspirin did not affect the production of this lipoxygenase product (Fig. 4c).

Overall, these data show that there is early platelet activation in sepsis which can be detected by quantification of monocyte-platelets aggregates in whole blood and soluble markers in plasma (sCD40L and eicosanoids).

### Sepsis induces a prothrombotic platelet state under flow and brings out an alternative mechanism enabling platelets to form stable thrombus at high shear rate in the absence of PI3Kβ

**Figure 5:**
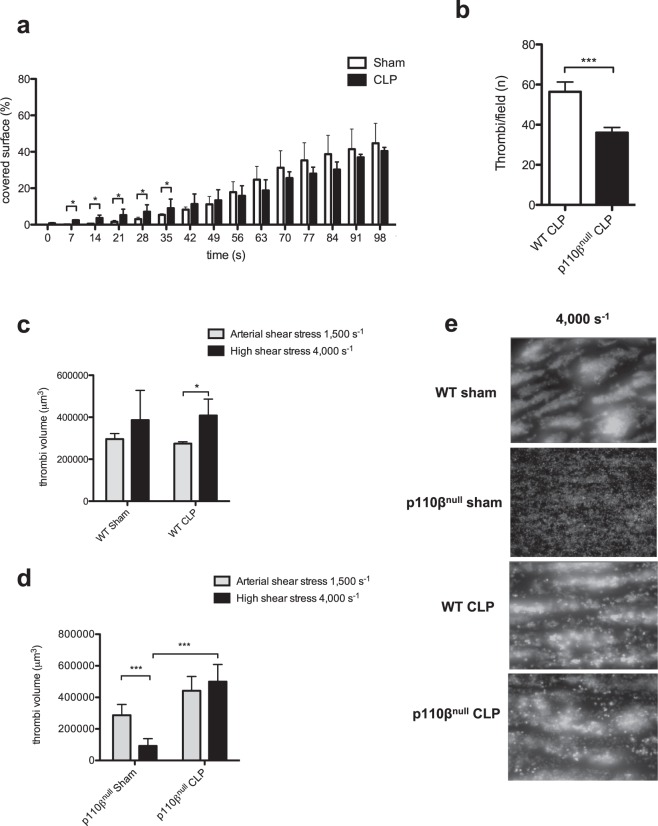
Platelet pro-thrombotic properties at arterial flow and bypass of PI3Kβ for thrombus stability at high shear rate during sepsis. (**a**) DIOC6-labeled platelets in whole blood from the CLP (black bar) or sham (white bar) mice 48 h post intervention were perfused through a collagen-coated microcapillary at a physiological arterial rate of 1500 s^−1^. Surface coverage (%) by fluorescent platelets was analyzed using ImageJ software. Results shown are median ± IQR of 4 independent experiments (*p < 0.05). (**b**) Platelet-rich thrombi formed in the liver 48 h post CLP were detected as in Fig. 2 (C-D) and quantified. 5 mice from each group and 5 to 10 field per mice were analyzed. Results are expressed as median ± IQR (***p < 0.001). (**c**–**e**) DIOC6-labeled platelets in whole blood from platelet PI3Kβ-deficient mice (p110β^null^) or wild type mice (WT) were perfused through a collagen-coated microcapillary at a physiological arterial shear rate of 1500 s^−1^, followed by a high shear rate of 4000 s^−1^. Thrombi volumes (μm^3^) were analyzed using ImageJ software. Results are expressed as median ± IQR of 4 to 6 independent experiments (***p < 0.001). (**e**) Representative images showing the platelet thrombi remaining after 1 min of high shear rate (4000 s^−1^).

To further investigate the effect of sepsis on platelets we studied thrombus formation under flow conditions encountered in microcirculation. Interestingly, despite a decreased platelet count at 48 hours, the CLP group exhibited a significantly faster adhesion and formation of platelet thrombus compared to the sham group of mice. However, after forty seconds of flow, the surface coverage was not significantly different in the two groups (Fig. 5a). Sepsis thus induced a pre-activation stage of platelets allowing them to very rapidly interact with the collagen surface at a shear rate of 1500 s^−1^ to form a growing thrombus.

Class I phosphoinositide 3-kinase β (PI3Kβ) is known to play an important role in platelet activation and has been proposed as a potential antithrombotic target^21,22,34^. To evaluate its role during sepsis we took advantage of our mouse model presenting an invalidation of the catalytic subunit of PI3Kβ (p110β) specifically in platelets^35^. As shown in Fig. 5b, following CLP, the number of thrombi in periportal zone of the liver was significantly decreased in the absence of PI3Kβ in platelets. However, this decrease had no effect on weight loss or the mean acute lung injury score 48 hours after CLP (Suppl Fig. 4). Deficiency of PI3Kβ in platelets is known to induce an instability in arterial thrombus at high shear rate^35^. Therefore, we performed CLP in wild type and p110β-deficient platelet mice and analyzed their capacity to form stable thrombi at high shear rate *ex-vivo*. Blood from both groups of mice was first perfused on collagen at 1500 s^−1^ for 150 seconds to form comparable thrombi and then an acceleration of the blood flow was generated to reach a high shear rate of 4000 s^−1^. In the wild type sham or CLP groups the thrombi formed at 1500 s^−1^ were stable and continued to grow at 4000 s^−1^ (Fig. 5c–e). As expected, in the absence of p110β, platelet thrombi rapidly destabilized at high shear rate, leaving a single platelet layer on the collagen surface with some small aggregates (Fig. 5d,e). Strikingly, septic conditions reversed the thrombus instability at high shear rate in p110β-deficient platelet mice. Indeed, following CLP, platelet thrombi were stable at 4000 s^−1^ indicating that sepsis allowed platelets to overcome the defect of thrombus stability induced by PI3Kβ invalidation at high shear rate (Fig. 5d,e).

## Discussion

Sepsis is a complex disease which can rapidly evolve to septic shock and subsequent organ failure in the absence of required early and aggressive management generally needed to stop the progression to septic shock and subsequent organ failure. Platelets are probably among the first responding cells during sepsis development and their roles and influences are likely different during sepsis progression. They can have protective roles by tempering macrophage-dependent inflammation, regulatory functions by maintaining inflammation or negative roles by precipitating microvessels dysfunction and in turn multiple organ failure. Here we analyzed the kinetics and characteristics of platelet activation and their behavior during sepsis in a mouse model of peritoneal sepsis^36^. CLP in rodents has become the most widely used model for experimental sepsis and considered a standard in sepsis research^37^. The survival rates found in our study, the severity of sepsis and multiple organ failure were comparable to those reported in the literature^36–38^. Lung histology revealed inflammatory acute lung injury as also reported in experimental polymicrobial peritonitis^36^. Thrombocytopenia was present significantly one day after CLP and persisted at 48 hours. Thrombocytopenia is common in Intensive Care Unit (ICU) patients and a decline in platelet count, even without thrombocytopenia, adds significant prognostic information to the current parameters used in the current ICU scoring system^39,40^. Several studies have reported that failure of the platelet count recovery to normal level during critical illness is associated with a higher mortality^41,42^.

The causes of thrombocytopenia in sepsis are still unclear but platelet consumption following activation and aggregation or adhesion to the endothelium and/or leukocytes likely contribute. Indeed, 48 hours after CLP we observed platelet-rich thrombi in the microcirculation of different organs including liver, kidney and lung. This appearance of platelet-rich thrombi correlated with organ failure and likely contributed to ischemic complications.

To study the kinetics of platelet activation during sepsis we analyzed different parameters including intrinsic and soluble platelet markers as well as formation of heterotypic platelet leukocytes aggregates. CD62P (P-selectin) was significantly increased at 24 hours post onset of sepsis. Consistent with this a significant increase in α_IIb_β_3_ activation assessed by fibrinogen binding on the platelet surface was observed 24 hours after CLP and persisted at 48 hours. In septic patients, Gawaz *et al*.^24^ also observed increased α_IIb_β_3_ activation on the platelet surface compared to controls. However, since activated platelets may rapidly be eliminated from circulation, measurement of intrinsic circulating platelet activation markers may not be sensitive enough to detect platelet activation, particularly in the first hours of sepsis^24^. Indeed, in patients with septic shock it has been suggested that the most active cells with high adhesion potential might be sequestered from circulation and thus escape detection^30,43^. In this context, we quantified soluble platelet markers and analyzed circulating heterotypic aggregates between platelets and leukocytes as valuable indicators of platelet activation during sepsis.

The formation of platelet-leukocyte aggregates is now recognized as a sensitive marker of *in vivo* platelet activation and is a feature of inflammatory conditions^30,44,45^. These aggregates are mediated via multiple ligands and receptors including P-selectin which is translocated to the surface of activated platelets^44^. We found an increase in monocyte-platelet aggregates formation, as early as 4 hours after CLP that persisted until 48 hours despite a drop in circulating monocyte count. The density of platelet per monocyte was also strongly increased. In contrast, the level of circulating neutrophil-platelet aggregates showed no significant differences until the first 24 hours but significantly increased 48 hours after CLP. An increase of platelet-leukocyte adhesion has been reported in patients with sepsis, and these heterotypic interactions were shown to decrease when patients developed multiple organ failure^24^. The exact mechanisms, regulations and clinical significance of such platelet-leukocyte interaction in sepsis are still poorly understood^46^. In our sepsis model the rapid increase in platelet-monocyte aggregates suggests early activation of a set of platelets in circulation. Consistent with this, the plasma level of sCD40L significantly increased 4 hours after CLP. These levels remained significantly high 8 hours after CLP but decreased 48 hours later. CD40L shedding from platelets following CLP has been shown to involve the matrix metalloprotease-9^47^. sCD40L contributes to the regulation of neutrophil recruitment and lung damage in sepsis. Higher sCD40L levels were found in 49 patients with meningococcal sepsis and 15 patients with African tick bite fever compared to controls^48,49^. In septic patients, a multicenter prospective study has shown that circulating sCD40L levels were significantly higher in septic patients than in controls, and in non-survivors compared to survivors^50^.

Activated platelets are known to generate several bioactive lipids including eicosanoids acting as regulators of a diverse set of homeostatic and inflammatory processes^33^. We found that circulating levels of the cyclo-oxygenase product TxB2 (the stable metabolite of TxA2) were significantly elevated in the early phase of sepsis and remained high 48 hours after CLP. As expected, treatment of mice with the cyclo-oxygenase inhibitor aspirin abolished this production. The lipo-oxygenase product 12-HETE was also significantly elevated 1 hour after CLP and was not affected by aspirin treatment. Overall, these data show that CLP-mediated experimental peritonitis in mice promotes a rapid activation of platelets detectable after 1 to 4 hours by measuring circulating soluble markers of platelet activation and monocyte-platelet aggregates. Activation markers at the surface of circulating platelets such as P-selectin expression and α_IIb_β_3_ activation are only significantly detectable 24 to 48 hours after CLP, when thrombocytopenia is already well established. Therefore, these results show that it is important to investigate both soluble and intrinsic platelet markers of activation to determinate the onset of platelet activation during sepsis.

Besides markers of platelet activation, another important question concerns the prothrombotic feature of platelets during sepsis, particularly at a shear rate encountered in microvessels. The dynamics of platelet thrombus formation and stabilization under flow is yet poorly studied in septic conditions. Using videomicroscopy analysis we observed that septic conditions significantly accelerated platelet adhesion and thrombus growth at a shear rate found in microvessels. Of note, this was observed with blood from mice 48 hours after CLP, a stage where a significant thrombocytopenia is present. This data reveals a prothrombotic behavior of platelets at arterial flow conditions during sepsis. Importantly, the thrombus formed were stable, even at very high shear rates. We and others have previously shown that PI3Kβ is mandatory for platelet activation and thrombus stability in both human and mouse models at high shear rate. PI3Kβ inhibitors have thus been proposed as potential antithrombotic drugs^21^. Therefore, we checked whether such inhibitors could be relevant to treat septic patients to prevent ischemic events arising from thrombosis in microcirculation, where the shear rate is elevated. Using p110β-null platelet mice, we found that absence of PI3Kβ significantly decreased the number of thrombi formed in the liver following CLP but was not sufficient to impair weight loss and lung injury. This decreased number of thrombi suggests that PI3Kβ contributed to the processes of platelet activation during sepsis. However, once formed these thrombi appeared stable at high shear rate even in the absence of PI3Kβ. In fact, sepsis restored the ability of platelets to form a stable thrombus at high shear rate in the absence of platelet PI3Kβ, indicating that septic conditions allow platelets to compensate for PI3Kβ deficiency. Thus, despite a decrease in the number of thrombi formed in the periportal zone of the liver, our results suggest that PI3Kβ inhibitors may not be sufficient to efficiently treat septic patients. How sepsis can modify platelets to allow them to form a stable thrombus at high shear rate in the absence of PI3Kβ remains to be established. It is noteworthy that the GpIb-VWF axis is exacerbated and important in sepsis^51–53^ and may contribute to compensate the lack of PI3Kβ in thrombus stabilization at high shear rates.

## Conclusions

Our results indicate that platelets are rapidly activated in the CLP model of peritonitis and that soluble and surface expression markers as well as monocyte-platelet aggregates should be quantified to determine platelet activation during sepsis. Thrombocytopenia can be attributed in part to platelet-rich thrombi formation in capillaries and platelet-leukocytes interactions. Platelets from septic mice have a prothrombotic phenotype at a shear rate encountered in arterioles. We propose that sepsis activates an alternative mechanism enabling platelets to bypass the normally mandatory role of PI3Kβ to form stable thrombus at high shear rates. Further studies are now necessary to unravel the molecular mechanisms leading to this prothrombotic state of platelets at high shear rate as this may unravel new risk markers and guide the development of future treatments of polymicrobial sepsis.

## Methods

### Animals

All animal procedures were in accordance with the guidelines of the Midi-Pyrénées Ethics Committee on Animal Experimentation (Comité National de Réflexion Ethique sur l′Expérimentation Animale – Midi-Pyrénées) and with the French Ministry of Agriculture license. This study was approved by the Midi-Pyrénées Ethics Committee on Animal Experimentation (N°MP/02/39/05/12, date 2012/10/02).

Male C57BL/6 J mice were obtained from Janvier Labs (Saint-Berthevin, Mayenne, France). PF4-cre/p110β^flox/flox^ mice were generated by crossing a mouse line in which exons 21 and 22 of the kinase domain of p110β are flanked by loxP sites (p110β flox/flox) with transgenic animals expressing the Cre recombinase specifically in megacaryocytes under the control of the PF4 promoter (PF4-Cre/p110wt/wt). PF4-Cre/p110β flox/wt mice were then crossed with p110β flox/flox mice to produce platelet-specific p110β-null mice PF4-Cre/110β flox/flox. They were obtained in a mendelian ratio, and were healthy, with no growth abnormalities. These animals exhibited normal size and platelet count^35,54^. We used 20-week-old animals, weighing 25–30 g. Mice had access to food and water *ad libitum* and were not fasted prior to CLP.

### Experimental protocol design of sepsis

Polymicrobial sepsis was induced by a CLP procedure as previously described^37,38^. Briefly, a laparotomy was performed under general anaesthesia, with the cecum ligated at 20% of its total length, below the ileocecal valve, and was punctured once with a 20-gauge needle. The cecum was then returned into the peritoneal cavity. Sham mice underwent the laparotomy without ligation and puncture. When indicated, mice were treated with aspirin (10 µg/g, intraperitoneal injection). Spontaneous mortality was followed four days after the surgery.

### Systemic platelet and leukocyte counts

Blood samples were collected by puncture of the lower vena cava utilizing heparinized syringe (0.1 ml heparin (100 IU/ml) for 0.9 ml of blood). Blood cells counts were performed on MICROS-60 analyzer (ABX-Diagnostics, Baden, Switzerland).

### Bacteriology

One milliliter of blood from sham and CLP group was cultured in Petri dishes with sheep blood and incubated for 48 hours at 37 °C in an aerobic atmosphere. Bacterial colonies identification was conducted using VITEK® automated system (BioMerieux, USA).

### Biochemical diagnostic assays

Serum was obtained after centrifugation (2800 *g*, 10 min) and immediately frozen and stored at −80 °C. Dosages were performed with PENTRA 400 ABX© (Horiba Medical ®) biochemical analyzer for serum creatinine, lactate dehydrogenase (LDH), aspartate aminotransferase (AST) and alanine aminotransferase (ALT).

### Flow cytometry

In order to analyze surface molecules expression (CD62P and α_IIb_β_3_) on circulating platelets, blood was collected (1:10 acid citrate dextrose (ACD: 3% trisodium citrate 5.5-hydrate, 1.4% citric acid, 2% anhydrous glucose)) at different time after surgery. Platelet Rich Plasma (PRP) was incubated (10 min, RT) with an anti-CD62P FITC-conjugated monoclonal antibody (BD Pharmigen^TM^) to analyze CD62P expression. PRP was also incubated with fluorescent fibrinogen (Fibrinogen Oregon green® 488, Invitrogen^TM^) permitting analysis of the changes in affinity of αIIbβ3 for fibrinogen. Platelet-leukocyte interactions were studied using blood samples (collected in 1:10 ACD), fixed prior to analysis with CELL-FIX© for 20 min and washed in 2 ml PBS. After centrifugation (300 *g*, 4 min), the pellet was resuspended and incubated with anti-CD41 FITC-conjugated monoclonal antibody (BD Pharmingen^TM^), PE-conjugated anti-Ly-6G Gr-1 monoclonal antibody, PE-conjugated anti-CD115 monoclonal antibody (eBioscience) or with the corresponding isotypes. ACK buffer (0.14 M ammonium chloride, 0.017 M Tris HCl, pH 7.4) was used for red blood cells lysis.

Samples were analyzed by flow cytometry using FACS-Verse^TM^ (BD Biosciences) and the FACS Suite^TM^ software. The platelet density per monocyte or neutrophil was evaluated by measuring the median fluorescence intensity (MFI) of the platelet marker (CD41) on monocytes or neutrophils as previously reported by Rutten *et al*.^31^.

### ELISA

Platelet poor plasma (PPP) was frozen in liquid nitrogen and stored at −80 °C before analysis. Soluble CD40 ligand (sCD40L) was measured in plasma with mouse sCD40L platinium ELISA kit (Affymetric eBioscience^TM^).

### Histology

Lungs were excised and formalin-fixed for 24 hours. Then, they were immersed in 70% ethanol solution. Four randomly selected sites from each inflation-fixed lung were embedded in paraffin, and sections were cut for hematoxylin and eosin staining. Lung injury severity was quantified in a blinded manner by adoption of a semi-quantitative scoring system, using the Acute Lung Injury (ALI) score taking into account 4 variables (leukocyte infiltration, fibrin/alveolar edema, alveolar wall thickness, intra-alveolar haemorrhage). The severity of each variable was quantified from 0 to 4 (absence, minimal: 1 to 3 alveoli, light > 3 alveoli, moderate: 2/3 of the surface area, severe: all the alveoli)^55^. Maximum score was 16. Leukocyte infiltration was studied by immunohistochemistry using the Ly6B.2 antibody (AbD Serotec^TM^ Bio-Rad Compagny).

### Plasma eicosanoids measurement by high performance liquid chromatography coupled to tandem mass spectrometry (LC-MS/MS)

PPP samples were stored at −80 °C until lipid extraction. LC-MS/MS was performed as detailed elsewhere^56^ using HPLC grade methanol, methyl formate and acetonitrile (Sigma-Aldrich). Briefly, lipid preparation from all samples was carried out through solid-phase extraction using hydrophobic polystyrene-divinylbenzene resin in dedicated 96-well plates (Chromabond multi96 HR-X 50 mg; Macherez-Nagel). After complete loading, columns were washed twice with H_2_O/MeOH (90/10, v/v) and dried under aspiration for 15 min. Samples were dried using nitrogen, dissolved again in methanol (10 μL) and transferred to liquid chromatography before LC-MS/MS analysis.

### Flow assays on collagen matrix

Biochips microcapillaries (Vena8Fluro + , Cellix) were coated with a collagen fibril suspension (50 μg/ml) and incubated at 37 °C for one hour prior to being saturated with a solution of 0.5% bovine serum albumin (BSA) in phosphate-buffered saline (PBS) without Ca^2+^/Mg^2+^. Mouse blood was drawn into heparin (10 IU/mL), and DIOC6 (2 µM) was used to label platelets. Using a syringe pump (Legato 200, KDScientifics) to apply a negative pressure, labeled blood was then perfused through a microcapillary for indicated time at a wall shear rate of 1500 seconds^−1^, and, when indicated, formed thrombi were then exposed to a high shear rate of 4000 seconds^−1^ as described^35^. Platelet adhesion and thrombus formation was visualized with a x40 oil immersion objective for both fluorescent and transmitted light microscopy; light source was provided by Colibri LED System (Zeiss) and was recorded (high resolution CCD cooled camera, Orca-R2, Hamamatsu) in real time (1 frame every 5 seconds). Image sequences of the time-lapse recording and analysis of surface coverage were performed offline on a single frame by quantification of pixel surface after manual thresholding using ImageJ. Thrombi volumes are calculated by thresholding of surface covered by thrombi on slice of Z-stack images and addition of voxel (automatically converted into µm^3^ by ZenZeiss software).

### Statistical analysis

Values are not normally distributed and are expressed as median and interquartile range (IQR). To compare differences between groups, the Mann-Whitney test was used. Analysis was performed using GraphPad Prism (version 5.0a for Mac). *P* value < 0.05 was considered significant and *n* represents the number of animals pooled together from each experiment.

## Electronic supplementary material

Supplementary Information

## Data Availability

The datasets used and/or analyzed during the current study are available from the corresponding author on reasonable request. All data generated or analyzed during this study are included in this published article and its supplementary information file.
