# Supplementary material for: Platelet activation and prothrombotic properties in a mouse model of peritoneal sepsis
Source: Sci Rep. 2018 Sep 10;8:13536. doi: 10.1038/s41598-018-31910-8 (PMC6131186; doi:10.1038/s41598-018-31910-8)
Supplement: Supplementary file 1 — Supplementary Information [file 41598_2018_31910_MOESM1_ESM.pdf]

PLATELET ACTIVATION AND PROTHROMBOTIC PROPERTIES IN A MOUSE MODEL OF PERITONEAL SEPSIS

Fanny Vardon Bounes, Vincent Mémier, Marina Marcaud, Aemilia Jacquemin, Hind Hamzeh-Cognasse, Cédric Garcia, Jennifer Series, Pierre Sié, Vincent Minville, Marie-Pierre Gratacap, Bernard Payrastre

Supplementary Figures

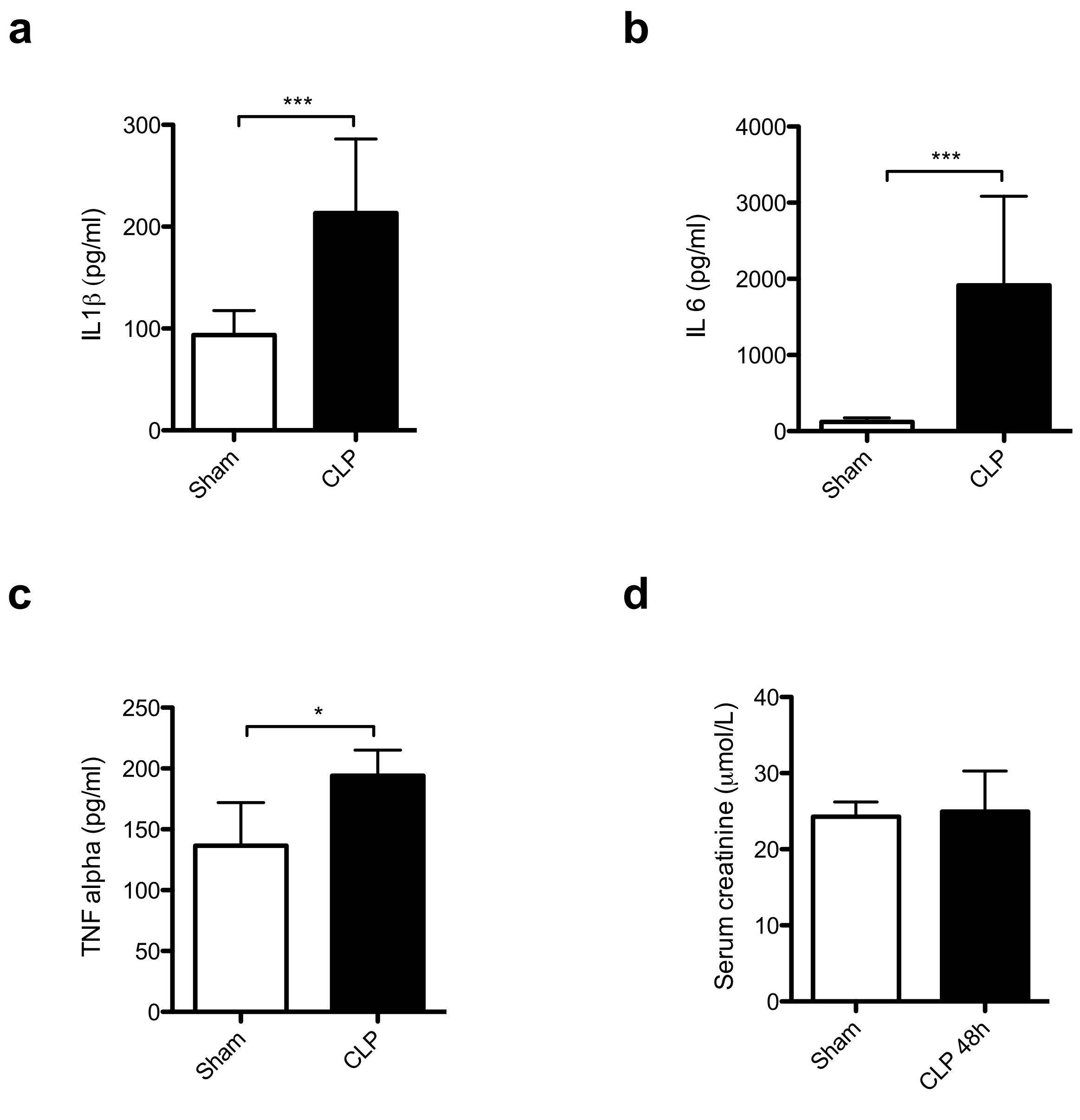

a
b
c
d
***
***
*
300
200
100
0
IL 1β (pg/ml)
4000
3000
2000
1000
0
IL 6 (pg/ml)
250
200
150
100
50
0
TNF alpha (pg/ml)
40
30
20
10
0
Serum creatinine (µmol/L)
Sham
CLP
Sham
CLP
Sham
CLP
Sham
CLP 48h

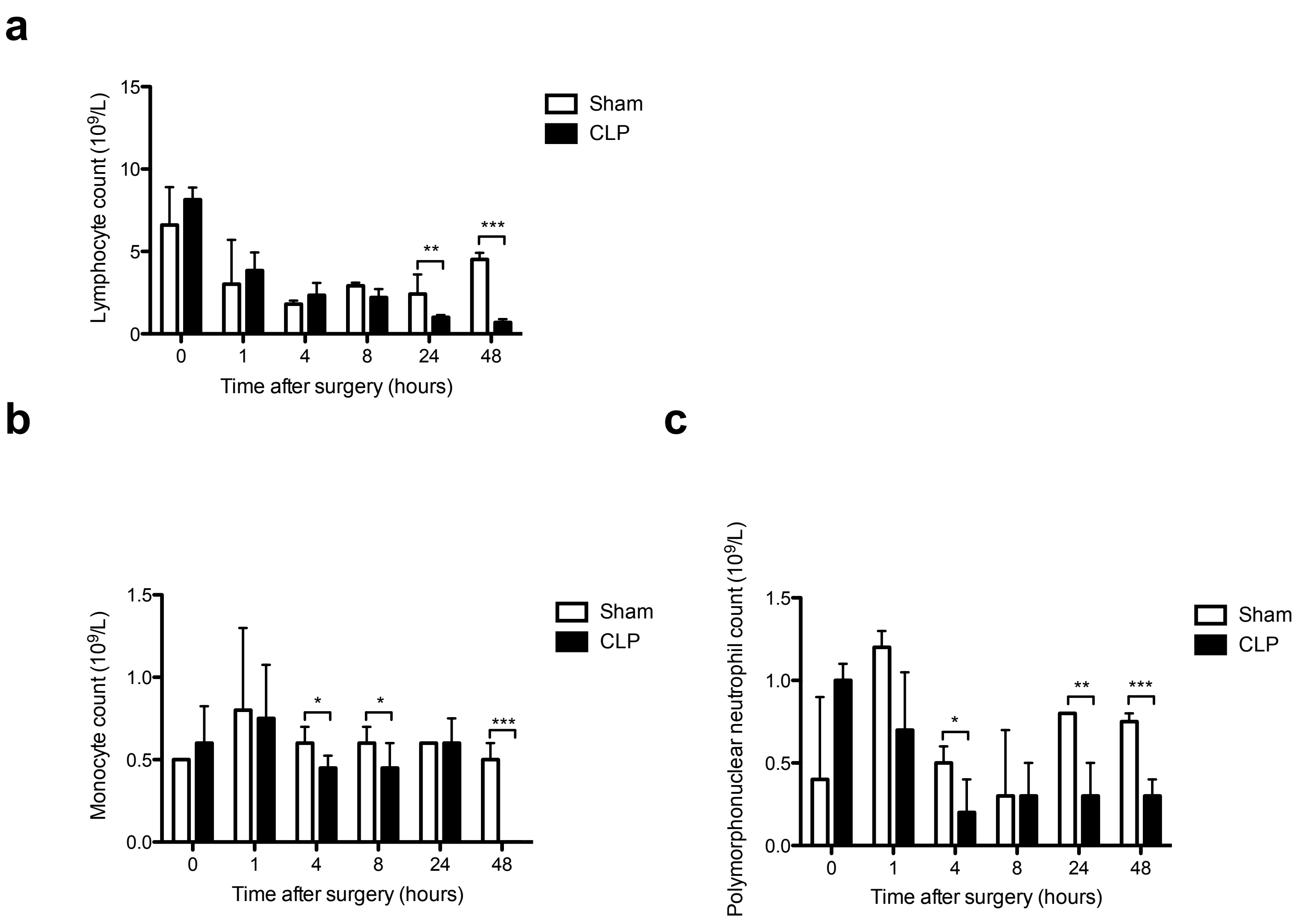
a
Lymphocyte count (10^9/L)
15
10
5
0
**
***
0
1
4
8
24
48
Time after surgery (hours)
Sham
CLP
b
Monocyte count (10^9/L)
1.5
1.0
0.5
0.0
*
*
***
0
1
4
8
24
48
Time after surgery (hours)
Sham
CLP
c
Polymorphonuclear neutrophil count (10^9/L)
1.5
1.0
0.5
0.0
*
**
***
0
1
4
8
24
48
Time after surgery (hours)
Sham
CLP

a

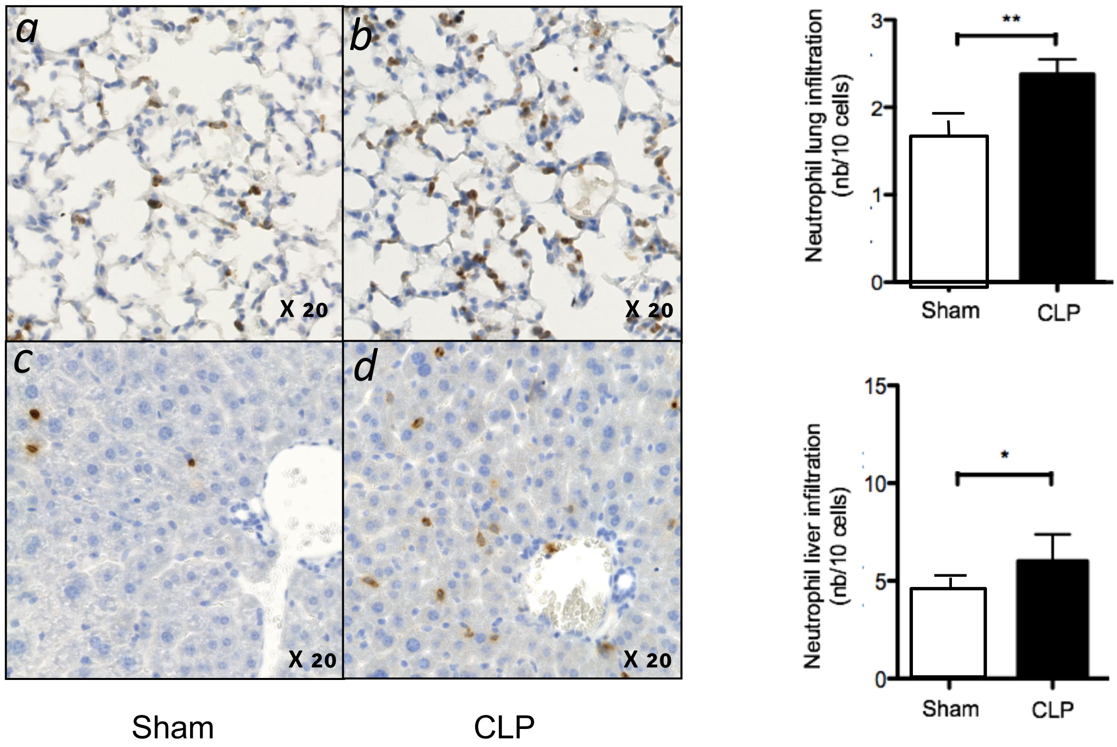
a
b
c
d
X 20
X 20
X 20
X 20
Sham
CLP
Neutrophil lung infiltration (nb/10 cells)
0
1
2
3
**
Sham
CLP
Neutrophil liver infiltration (nb/10 cells)
0
5
10
15
*
Sham
CLP

b

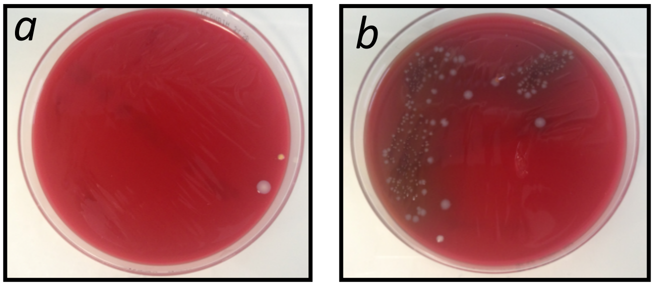
a
b

a

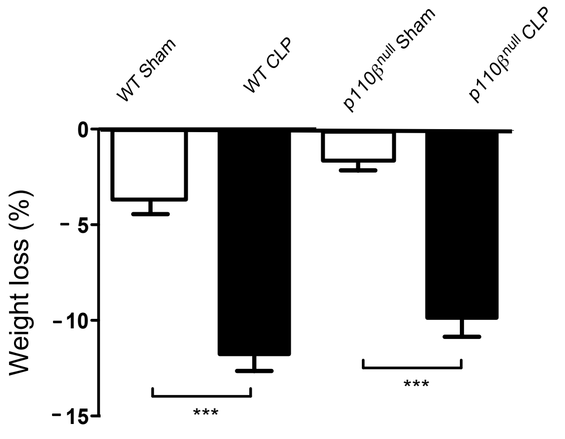

WT Sham
WT CLP
p110β^null Sham
p110β^null CLP
0
- 5
-10
-15
Weight loss (%)
***
***

b

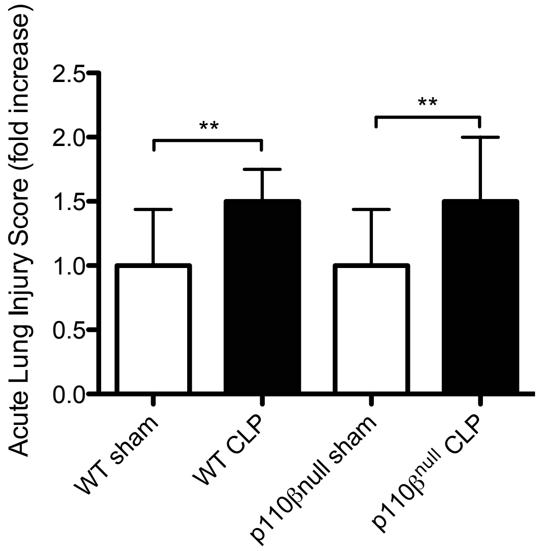

Acute Lung Injury Score (fold increase)
2.5
2.0
1.5
1.0
0.5
0.0
**
**
WT sham
WT CLP
p110βnull sham
p110β^null CLP

Supplementary figures legends

Supplementary Figure 1. Influence of sepsis on cytokines plasmatic levels serum creatinine level. Comparison of plasma levels of (**a**) IL1β, (**b**) IL6 and (**c**) TNFα 6 h post CLP. (**d**) Serum creatinine analysis was performed with a PENTRA 400 ABXc analyzer. Whisker boxes are presented as follows: min, max, median, 25-75th percentiles. Results are expressed as median ± IQR of 10 independent experiments (*$p<0.05$, ***$p<0.001$).

Supplementary Figure 2. Influence of sepsis on leukocyte counts after the onset of the infection. Comparison lymphocytes (**a**), monocytes (**b**) and neutrophils (**c**) kinetics counts in sham and CLP-operated mice. Results are expressed as G/L and are median ± IQR of 6 to 30 independent experiments (*$p<0.05$, **$p<0.01$, ***$p<0.001$).

Supplementary Figure 3. Influence of sepsis on leukocyte infiltration and identification of bacteria from blood culture. (**a**) Representative histological sections of lung (*a,b*) and liver (*c,d*) tissues 48 h post surgery. Sections from CLP (*b, d*) or sham-operated animals (*a,c*) as controls were stained with Masson's trichrome and neutrophils were specifically labeled with an anti-Ly6b antibody. Images (x20 magnification) and histograms shown are representative of 3 independent experiments (* $p<0.05$, ** $p<0.01$). (**b**) Comparison of bacteria blood culture from sham (*a*) and CLP-operated (*b*) mice. Blood bacteria were cultured in Petri dishes with sheep blood and incubated for 48 h at 37°C in an aerobic atmosphere. Microbial identification was done using the Biomérieux Vitek® 2 system (Hazelwood, MO, USA), an automated microbiology system utilizing growth-based technology, using colorimetric reagent cards that are incubated with the microorganism and interpreted automatically. *Enterococcus faecalis,* a gram positive bacteria, and *citrobacter braakii,* a gram negative bacteria were identified.

Supplementary Figure 4. Lack of PI3Kβ in platelets does not affect weight loss and lung injury during sepsis. (a) Weight loss was measured 48 h post procedure in the sham-operated or the CLP groups of wild type (WT) or platelet PI3Kβ-deficient ($p110\beta^{null}$) mice. Results are expressed as percentage of weight loss and are median ± IQR (n=8 to 13, ***$p<0.001$). **(b)** The Acute Lung Injury (ALI) Score was quantified as in figure 1H in the sham-operated or the CLP group of wild type (WT) or platelet PI3Kβ-deficient ($p110b^{null}$) mice. Histograms shown are representative of 4 independent experiments (**$p<0.01$).
